# 3,3′-(Ethane-1,2-di­yl)bis­(3,4-dihydro-2*H*-1,3-benzoxazine)

**DOI:** 10.1107/S1600536811053530

**Published:** 2011-12-17

**Authors:** Augusto Rivera, Jairo Camacho, Jaime Ríos-Motta, Karla Fejfarová, Michal Dušek

**Affiliations:** a Departamento de Química, Universidad Nacional de Colombia, Ciudad Universitaria, Bogotá, Colombia; b Institute of Physics ASCR, v.v.i., Na Slovance 2, 182 21 Praha 8, Czech Republic

## Abstract

The title compound, C_18_H_20_N_2_O_2_, was prepared by Mannich-type reaction of phenol, ethane-1,2-diamine and formaldehyde. The heterocyclic rings adopt half-chair conformations. The acyclic methyl­ene groups attached to the N atoms are in an axial position. In the crystal, weak C—H⋯O hydrogen bonds link the mol­ecules into dimers. These dimers are further connected *via* C—H⋯π contacts.

## Related literature

For related structures see: Rivera *et al.* (2011 ▶, 2010 ▶). For the preparation of the title compound, see: Rivera *et al.* (1989 ▶). For ring conformations, see Cremer & Pople (1975 ▶). For weak hydrogen bonds, see: Desiraju & Steiner (1999 ▶).
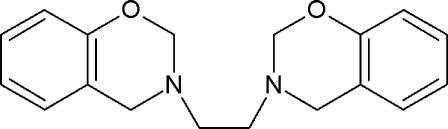


## Experimental

### 

#### Crystal data

C_18_H_20_N_2_O_2_
*M* *_r_* = 296.4
Monoclinic, 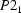

*a* = 10.868 (2) Å
*b* = 5.1693 (13) Å
*c* = 13.327 (3) Å
β = 102.623 (18)°
*V* = 730.6 (3) Å^3^
*Z* = 2
Cu *K*α radiation
μ = 0.71 mm^−1^
*T* = 120 K
0.97 × 0.10 × 0.04 mm

#### Data collection

Agilent Xcalibur diffractometer with an Atlas (Gemini ultra Cu) detector
Absorption correction: multi-scan (*CrysAlis PRO*; Agilent, 2010 ▶) *T*_min_ = 0.77, *T*_max_ = 1
2799 measured reflections
1341 independent reflections
785 reflections with *I* > 3σ(*I*)
*R*_int_ = 0.079

#### Refinement

*R*[*F* ^2^ > 2σ(*F* ^2^)] = 0.067
*wR*(*F* ^2^) = 0.171
*S* = 1.38
1341 reflections
199 parameters
H-atom parameters constrained
Δρ_max_ = 0.28 e Å^−3^
Δρ_min_ = −0.25 e Å^−3^

### 

Data collection: *CrysAlis PRO* (Agilent, 2010 ▶); cell refinement: *CrysAlis PRO*; data reduction: *CrysAlis PRO*; program(s) used to solve structure: *SIR2002* (Burla *et al.*, 2003 ▶); program(s) used to refine structure: *JANA2006* (Petříček *et al.*, 2006 ▶); molecular graphics: *DIAMOND* (Brandenburg & Putz, 2005 ▶); software used to prepare material for publication: *JANA2006*.

## Supplementary Material

Crystal structure: contains datablock(s) global, I. DOI: 10.1107/S1600536811053530/bt5748sup1.cif

Structure factors: contains datablock(s) I. DOI: 10.1107/S1600536811053530/bt5748Isup2.hkl

Supplementary material file. DOI: 10.1107/S1600536811053530/bt5748Isup3.cml

Additional supplementary materials:  crystallographic information; 3D view; checkCIF report

## Figures and Tables

**Table 1:** Hydrogen-bond geometry (Å, °) *Cg*4 is the centroid of the C12–C17 ring.

*D*—H⋯*A*	*D*—H	H⋯*A*	*D*⋯*A*	*D*—H⋯*A*
C11—H11*a*⋯O2^i^	0.96	2.47	3.415 (10)	168
C11—H11*b*⋯*Cg*4^ii^	0.96	2.58	3.523 (10)	169

Symmetry codes: (i) 
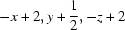
; (ii) 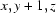.

**Fig. 1.**
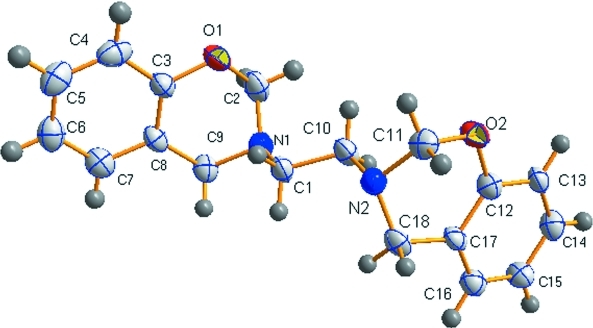
A view of (I) with the numbering scheme.displacement ellipsoids are drawn at the 50% probability level.

**Fig. 2.**
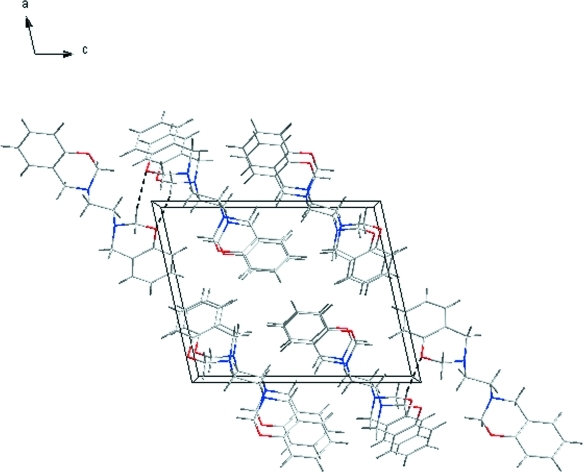
Packing of the molecules of the title compound view along b axis.

## supplementary crystallographic information

### Comment

We have recently reported the molecular structure of two 3,3'-(ethane-1,2-diyl)bis(6-substituted-3,4-dihydro-2*H*-1,3-benzoxazine). The substituents in position 6 were methyl and chlorine respectively (Rivera *et al.*, 2011, 2010). Their crystal structures established the existence of an anomeric effect in N—C—O sequence in oxazine ring. In connection with our interest in anomeric effect in benzo-fused oxazine ring, we decided it was important to establish the effect of substituent at the aromatic ring in the N—C—O moiety. Thus, we obtained the title compound (I) which has no substituent in position 6.

The molecular structure of the title compound is illustrated in Fig. 1. Unlike the related structures, which crystallized in monoclinic space groups *P*2_1_*/n* (Rivera *et al.*, 2011) and C2/*c* (Rivera *et al.*, 2010) utilizing the crystallography inversion center in the molecular symmetry, the title compound (I) crystallizes in the polar space group *P*2_1_ with one molecule in the asymmetric unit. The molecules of (I) thus have no internal symmetry. The fused six-membered heterocyclic rings exists in the approximate half-chair conformations with puckering parameters Q = 0.479 (9) Å, θ = 49.2 (11)° and φ = 94.4 (13)° for O1/C2/N1/C9/C8/C3 and Q = 0.482 (8) Å, θ = 50.0 (10)° and φ = 101.1 (13)° for O2/C11/N2/C18/C17/C12 (Cremer & Pople, 1975). The C—O bond lengths [C2—O1, 1.451 (13) Å; C11—O2, 1.475 (11) Å] are longer than the values observed in related structure where the *p*-substituents in the aromatic rings is methyl [1.3755 (14) Å and 1.4525 (13) Å] (Rivera *et al.*, 2011). However, in *p*-chlorine derivative, the C—O bond distance is significantlly longer from those in (I), [1.421 (2) Å and 1.529 (2) Å] (Rivera *et al.*, 2010). The N1—C2 and N2—C11 bond lengths of 1.416 (9) Å and 1.431 (10) Å respectively, which are shorter than the expected bond length of 1.468 Å, provides structural evidence for the existence of an anomeric effect in both N—C—O groups.

In the crystal weak intermolecular C—H···O contacts (Table 1) that could be considered as weak hydrogen bonds (Desiraju & Steiner, 1999) link molecules into dimers (Fig. 2). Neighboring pair of these dimers are linked together *via* weaker C—H···π contacts into chains extended along the *b* axis (Figure 2).

### Experimental

To a stirred mixture of ethane-1,2-diamine (0.34 ml, 5 mmol) and phenol (0.94 g, 10 mmol) dissolved in dioxane (10 ml) was added dropwise an aqueous solution of formaldehyde (1.5 ml, 20 mmol). The reaction mixture was stirred for4 h. at room temperature. The resultant precipitate was collected, washed with water, dried in vacuum and recrystallized from ethanol to give title compound.

### Refinement

All H atoms atoms were positioned geometrically and treated as riding on their parent atoms. The isotropic atomic displacement parameters of hydrogen atoms were evaluated as 1.2×*U*_eq_ of the parent atom. As the structure contains only light atoms, the Friedel-pair reflections were merged and the Flack parameter has not been determined.

### Crystal data

**Table:**

C_18_H_20_N_2_O_2_	*F*(000) = 316
*M**_r_* = 296.4	*D*_x_ = 1.347 Mg m^−^^3^
Monoclinic, *P*2_1_	Cu *K*α radiation, λ = 1.5418 Å
Hall symbol: P 2yb	Cell parameters from 1061 reflections
*a* = 10.868 (2) Å	θ = 3.4–65.5°
*b* = 5.1693 (13) Å	µ = 0.71 mm^−^^1^
*c* = 13.327 (3) Å	*T* = 120 K
β = 102.623 (18)°	Needle, colourless
*V* = 730.6 (3) Å^3^	0.97 × 0.10 × 0.04 mm
*Z* = 2	

### Data collection

**Table:**

Agilent Xcalibur diffractometer with an Atlas (Gemini ultra Cu) detector	1341 independent reflections
Radiation source: Enhance Ultra (Cu) X-ray Source	785 reflections with *I* > 3σ(*I*)
mirror	*R*_int_ = 0.079
Detector resolution: 10.3784 pixels mm^-1^	θ_max_ = 65.7°, θ_min_ = 3.4°
Rotation method data acquisition using ω scans	*h* = −12→9
Absorption correction: multi-scan (*CrysAlis PRO*; Agilent, 2010)	*k* = −4→5
*T*_min_ = 0.77, *T*_max_ = 1	*l* = −15→15
2799 measured reflections	

### Refinement

**Table:**

Refinement on *F*^2^	81 constraints
*R*[*F*^2^ > 2σ(*F*^2^)] = 0.067	H-atom parameters constrained
*wR*(*F*^2^) = 0.171	Weighting scheme based on measured s.u.'s *w* = 1/[σ^2^(*I*) + 0.0016*I*^2^]
*S* = 1.38	(Δ/σ)_max_ = 0.004
1341 reflections	Δρ_max_ = 0.28 e Å^−^^3^
199 parameters	Δρ_min_ = −0.25 e Å^−^^3^
0 restraints	

### Special details

**Table:**

Experimental. CrysAlisPro (Agilent, 2010) Empirical absorption correction using spherical harmonics, implemented in SCALE3 ABSPACK scaling algorithm.
Refinement. The refinement was carried out against all reflections. The conventional *R*-factor is always based on *F*. The goodness of fit as well as the weighted *R*-factor are based on *F* and *F*^2^ for refinement carried out on *F* and *F*^2^, respectively. The threshold expression is used only for calculating *R*-factors *etc*. and it is not relevant to the choice of reflections for refinement.The program used for refinement, Jana2006, uses the weighting scheme based on the experimental expectations, see _refine_ls_weighting_details, that does not force *S* to be one. Therefore the values of *S* are usually larger than the ones from the *SHELX* program.

### Fractional atomic coordinates and isotropic or equivalent isotropic displacement parameters (Å^2^)

**Table:**

	*x*	*y*	*z*	*U*_iso_*/*U*_eq_	
C1	0.9998 (6)	0.619 (2)	0.7044 (5)	0.038 (2)	
N1	1.0853 (5)	0.4006 (19)	0.7006 (4)	0.040 (2)	
C2	1.2051 (7)	0.420 (2)	0.7681 (6)	0.049 (3)	
O1	1.2790 (5)	0.6444 (18)	0.7528 (4)	0.0482 (19)	
C3	1.2853 (7)	0.677 (2)	0.6506 (5)	0.039 (3)	
C4	1.3740 (7)	0.854 (2)	0.6314 (6)	0.050 (3)	
C5	1.3841 (7)	0.898 (2)	0.5301 (6)	0.051 (3)	
C6	1.3057 (7)	0.766 (2)	0.4522 (6)	0.049 (3)	
C7	1.2171 (7)	0.595 (2)	0.4717 (6)	0.046 (3)	
C8	1.2051 (7)	0.549 (2)	0.5724 (5)	0.036 (2)	
C9	1.1083 (7)	0.361 (2)	0.5963 (5)	0.040 (3)	
C10	0.9615 (6)	0.630 (2)	0.8071 (5)	0.039 (2)	
N2	0.8679 (5)	0.8366 (19)	0.8073 (4)	0.036 (2)	
C11	0.8763 (7)	0.948 (2)	0.9069 (5)	0.039 (3)	
O2	0.8508 (4)	0.7615 (18)	0.9838 (3)	0.0389 (16)	
C12	0.7491 (7)	0.605 (2)	0.9472 (5)	0.036 (2)	
C13	0.7116 (6)	0.445 (2)	1.0211 (5)	0.037 (2)	
C14	0.6127 (7)	0.273 (2)	0.9901 (6)	0.043 (3)	
C15	0.5531 (7)	0.262 (2)	0.8873 (5)	0.039 (2)	
C16	0.5905 (6)	0.416 (2)	0.8150 (5)	0.040 (3)	
C17	0.6897 (6)	0.589 (2)	0.8434 (5)	0.033 (2)	
C18	0.7386 (6)	0.756 (2)	0.7673 (5)	0.035 (2)	
H1a	0.925907	0.601938	0.65019	0.0451*	
H1b	1.040864	0.777887	0.693451	0.0451*	
H2a	1.252659	0.265585	0.763506	0.0584*	
H2b	1.195273	0.416764	0.837985	0.0584*	
H4	1.427396	0.94568	0.687131	0.0595*	
H5	1.444994	1.018264	0.5152	0.0611*	
H6	1.313009	0.793375	0.382469	0.0593*	
H7	1.163036	0.505915	0.415763	0.0546*	
H9a	1.030594	0.380027	0.546277	0.0478*	
H9b	1.136814	0.186835	0.590176	0.0478*	
H10a	0.926386	0.466841	0.820454	0.0473*	
H10b	1.034626	0.662279	0.860732	0.0473*	
H11a	0.958427	1.021981	0.930714	0.0474*	
H11b	0.818703	1.090401	0.901728	0.0474*	
H13	0.754437	0.454456	1.092073	0.0449*	
H14	0.585732	0.163708	1.039393	0.0519*	
H15	0.484253	0.143471	0.865763	0.0472*	
H16	0.547427	0.403116	0.744117	0.0481*	
H18a	0.733488	0.661676	0.70447	0.0422*	
H18b	0.685945	0.905848	0.750886	0.0422*	

### Atomic displacement parameters (Å^2^)

**Table:**

	*U*^11^	*U*^22^	*U*^33^	*U*^12^	*U*^13^	*U*^23^
C1	0.041 (4)	0.046 (4)	0.027 (3)	0.005 (4)	0.010 (3)	0.005 (3)
N1	0.042 (3)	0.041 (3)	0.038 (4)	0.008 (3)	0.012 (3)	0.002 (3)
C2	0.058 (5)	0.053 (5)	0.037 (4)	0.011 (5)	0.013 (4)	0.010 (4)
O1	0.049 (3)	0.064 (3)	0.029 (3)	0.002 (3)	0.003 (2)	−0.004 (3)
C3	0.044 (5)	0.044 (4)	0.029 (4)	−0.002 (4)	0.008 (3)	−0.001 (3)
C4	0.039 (4)	0.050 (5)	0.059 (6)	−0.001 (4)	0.009 (4)	−0.017 (4)
C5	0.053 (5)	0.035 (4)	0.068 (6)	−0.005 (4)	0.022 (4)	−0.006 (4)
C6	0.060 (5)	0.040 (4)	0.053 (5)	0.003 (5)	0.023 (4)	0.004 (4)
C7	0.054 (5)	0.040 (4)	0.043 (4)	0.002 (4)	0.010 (4)	−0.001 (4)
C8	0.045 (4)	0.034 (4)	0.028 (4)	0.004 (4)	0.009 (3)	−0.002 (3)
C9	0.047 (5)	0.035 (4)	0.040 (4)	−0.002 (3)	0.014 (3)	−0.004 (3)
C10	0.048 (4)	0.044 (4)	0.025 (4)	0.006 (4)	0.006 (3)	0.003 (3)
N2	0.036 (3)	0.041 (3)	0.033 (4)	0.003 (3)	0.010 (3)	0.002 (3)
C11	0.042 (4)	0.034 (4)	0.042 (4)	−0.003 (4)	0.008 (3)	0.004 (3)
O2	0.046 (3)	0.036 (2)	0.032 (3)	−0.008 (3)	0.004 (2)	−0.001 (2)
C12	0.039 (4)	0.033 (4)	0.035 (4)	0.000 (4)	0.009 (3)	−0.001 (3)
C13	0.043 (4)	0.046 (4)	0.024 (4)	0.003 (4)	0.011 (3)	−0.003 (3)
C14	0.050 (5)	0.041 (4)	0.041 (4)	−0.001 (4)	0.016 (3)	−0.004 (4)
C15	0.044 (4)	0.035 (4)	0.039 (4)	−0.004 (4)	0.008 (3)	−0.001 (3)
C16	0.043 (4)	0.042 (4)	0.035 (4)	0.004 (4)	0.008 (3)	−0.004 (3)
C17	0.038 (4)	0.039 (4)	0.022 (3)	0.003 (4)	0.005 (3)	−0.007 (3)
C18	0.046 (4)	0.030 (3)	0.028 (4)	0.008 (4)	0.006 (3)	0.006 (3)

### Geometric parameters (Å, °)

**Table:**

C1—N1	1.471 (13)	C10—N2	1.474 (12)
C1—C10	1.516 (10)	C10—H10a	0.96
C1—H1a	0.96	C10—H10b	0.96
C1—H1b	0.96	N2—C11	1.431 (10)
N1—C2	1.416 (9)	N2—C18	1.450 (9)
N1—C9	1.480 (10)	C11—O2	1.475 (11)
C2—O1	1.451 (13)	C11—H11a	0.96
C2—H2a	0.96	C11—H11b	0.96
C2—H2b	0.96	O2—C12	1.370 (10)
O1—C3	1.388 (9)	C12—C13	1.415 (12)
C3—C4	1.394 (13)	C12—C17	1.395 (9)
C3—C8	1.373 (11)	C13—C14	1.386 (12)
C4—C5	1.397 (12)	C13—H13	0.96
C4—H4	0.96	C14—C15	1.383 (9)
C5—C6	1.373 (12)	C14—H14	0.96
C5—H5	0.96	C15—C16	1.378 (12)
C6—C7	1.373 (13)	C15—H15	0.96
C6—H6	0.96	C16—C17	1.390 (12)
C7—C8	1.397 (11)	C16—H16	0.96
C7—H7	0.96	C17—C18	1.514 (12)
C8—C9	1.518 (13)	C18—H18a	0.96
C9—H9a	0.96	C18—H18b	0.96
C9—H9b	0.96		
			
N1—C1—C10	111.1 (7)	C1—C10—N2	110.8 (7)
N1—C1—H1a	109.4711	C1—C10—H10a	109.4713
N1—C1—H1b	109.4709	C1—C10—H10b	109.4708
C10—C1—H1a	109.4717	N2—C10—H10a	109.4714
C10—C1—H1b	109.4713	N2—C10—H10b	109.4714
H1a—C1—H1b	107.7691	H10a—C10—H10b	108.1247
C1—N1—C2	115.1 (8)	C10—N2—C11	112.8 (6)
C1—N1—C9	112.2 (6)	C10—N2—C18	113.9 (8)
C2—N1—C9	106.6 (6)	C11—N2—C18	108.4 (6)
N1—C2—O1	115.3 (7)	N2—C11—O2	113.5 (8)
N1—C2—H2a	109.4712	N2—C11—H11a	109.4711
N1—C2—H2b	109.4708	N2—C11—H11b	109.4713
O1—C2—H2a	109.4715	O2—C11—H11a	109.4716
O1—C2—H2b	109.4712	O2—C11—H11b	109.4711
H2a—C2—H2b	102.9256	H11a—C11—H11b	105.115
C2—O1—C3	112.5 (7)	C11—O2—C12	113.3 (5)
O1—C3—C4	116.4 (7)	O2—C12—C13	115.5 (6)
O1—C3—C8	121.8 (8)	O2—C12—C17	123.5 (8)
C4—C3—C8	121.8 (7)	C13—C12—C17	120.9 (8)
C3—C4—C5	119.1 (8)	C12—C13—C14	119.4 (6)
C3—C4—H4	120.4314	C12—C13—H13	120.2967
C5—C4—H4	120.4309	C14—C13—H13	120.2963
C4—C5—C6	119.0 (9)	C13—C14—C15	119.2 (8)
C4—C5—H5	120.5111	C13—C14—H14	120.4096
C6—C5—H5	120.5112	C15—C14—H14	120.4094
C5—C6—C7	121.4 (8)	C14—C15—C16	121.5 (8)
C5—C6—H6	119.2825	C14—C15—H15	119.2499
C7—C6—H6	119.2824	C16—C15—H15	119.2504
C6—C7—C8	120.5 (7)	C15—C16—C17	120.8 (6)
C6—C7—H7	119.7553	C15—C16—H16	119.6195
C8—C7—H7	119.7559	C17—C16—H16	119.6206
C3—C8—C7	118.1 (8)	C12—C17—C16	118.3 (8)
C3—C8—C9	120.3 (7)	C12—C17—C18	118.4 (7)
C7—C8—C9	121.6 (7)	C16—C17—C18	123.4 (6)
N1—C9—C8	112.0 (7)	N2—C18—C17	111.8 (5)
N1—C9—H9a	109.4708	N2—C18—H18a	109.4715
N1—C9—H9b	109.4706	N2—C18—H18b	109.4717
C8—C9—H9a	109.4711	C17—C18—H18a	109.471
C8—C9—H9b	109.4722	C17—C18—H18b	109.4706
H9a—C9—H9b	106.8364	H18a—C18—H18b	107.0112
			
?—?—?—?	?		

### Hydrogen-bond geometry (Å, °)

**Table:**

Cg4 is the centroid of the C12–C17 ring.

**Table:**

*D*—H···*A*	*D*—H	H···*A*	*D*···*A*	*D*—H···*A*
C11—H11a···O2^i^	0.96	2.47	3.415 (10)	168.
C11—H11b···Cg4^ii^	0.96	2.58	3.523 (10)	169

Symmetry codes: (i) −*x*+2, *y*+1/2, −*z*+2; (ii) *x*, *y*+1, *z*.
